# In vitro ultraviolet–induced damage in human corneal, lens, and retinal pigment epithelial cells

**Published:** 2011-01-21

**Authors:** Hyun-Yi Youn, David J. McCanna, Jacob G. Sivak, Lyndon W. Jones

**Affiliations:** School of Optometry, University of Waterloo, Waterloo, Ontario, Canada

## Abstract

**Purpose:**

The purpose was to develop suitable in vitro methods to detect ocular epithelial cell damage when exposed to UV radiation, in an effort to evaluate UV-absorbing ophthalmic biomaterials.

**Methods:**

Human corneal epithelial cells (HCEC), lens epithelial cells (HLEC), and retinal pigment epithelial cells (ARPE-19) were cultured and Ultraviolet A/Ultraviolet B (UVA/UVB) blocking filters and UVB-only blocking filters were placed between the cells and a UV light source. Cells were irradiated with UV radiations at various energy levels with and without filter protections. Cell viability after exposure was determined using the metabolic dye alamarBlue and by evaluating for changes in the nuclei, mitochondria, membrane permeability, and cell membranes of the cells using the fluorescent dyes Hoechst 33342, rhodamine 123, calcein AM, ethidium homodimer-1, and annexin V. High-resolution images of the cells were taken with a Zeiss 510 confocal laser scanning microscope.

**Results:**

The alamarBlue assay results of UV-exposed cells without filters showed energy level-dependent decreases in cellular viability. However, UV treated cells with 400 nm LP filter protection showed the equivalent viability to untreated control cells at all energy levels. Also, UV irradiated cells with 320 nm LP filter showed lower cell viability than the unexposed control cells, yet higher viability than UV-exposed cells without filters in an energy level-dependent manner. The confocal microscopy results also showed that UV radiation can cause significant dose-dependent degradations of nuclei and mitochondria in ocular cells. The annexin V staining also showed an increased number of apoptotic cells after UV irradiation.

**Conclusions:**

The findings suggest that UV-induced HCEC, HLEC, and ARPE-19 cell damage can be evaluated by bioassays that measure changes in the cell nuclei, mitochondria, cell membranes, and cell metabolism, and these assay methods provide a valuable in vitro model for evaluating the effectiveness of UV-absorbing ophthalmic biomaterials, including contact lenses and intraocular lenses.

## Introduction

Ultraviolet (UV) radiation from sunlight is commonly divided into two components. These components are UVB (290–320 nm) and UVA (320–400 nm). Exposure to UVB and UVA radiation is associated with photochemical damage to cellular systems. For example, UV radiation can generate free radicals including oxygen-derived species [1], which are known to cause lipid peroxydation of cellular membranes [1]. It has also been shown that UV can damage DNA directly [2,3], decrease mitochondrial function [4], and induce apoptosis [5]. There are three critical ocular structures that could be affected by UV exposure: the cornea, the lens, and the retina. The cornea transmits radiant energy only at 295 nm and above [6]. The crystalline lens absorbs almost all incident energy to wavelengths of nearly 400 nm [6]. Oblique rays entering the eye from the temporal side, can reach the equatorial (germinative) area of the lens.

There are intraocular filters that effectively filter different parts of the UV spectrum and only allow 1% or less to reach the retina [7]. Nevertheless, this small fraction of energy, if phototoxic, could still be of concern [7]. Furthermore, the removal of the lens by cataract surgery, which is one of the most commonly performed surgeries worldwide, may be associated with a substantial increase in the UV radiation that reaches the retina if the intraocular lens does not block UV appropriately. Chronic exposure to UV radiation may play a significant contributory role in the development of eye diseases, such as photokeratitis, pterygium, pinguecula, cataract, and macular degeneration [8]. Primary ocular defense strategies against these ill effects solely relate to the recessed location of the eye in the orbit and partial closing of the eyelids in response to high visible light intensities. Sunglasses and shading hats do not provide complete protection from scattered and incident UV light [9].

UV-absorbing ophthalmic biomaterials, such as contact lenses and intraocular lenses, have been available for increasing protection of the internal structure of the eye. Until recently, the majority of soft contact lenses were manufactured with negligible UV absorbing capability. Also, class I UV-absorbing silicone hydrogel polymers have been introduced most recently, and, to date, little has been published on the UV-attenuating properties of silicone hydrogel contact lenses [10,11]. Most intraocular lenses incorporate UV blocking chromophores, but several intraocular lenses currently in use exhibit inadequate light-absorbing properties [12,13]. Thus, there is a need to evaluate and compare the performance of these UV-absorbing ophthalmic biomaterials. While there are many studies that evaluate only the spectral transmission characteristics of contact lenses or intraocular lenses to verify their anti-UV efficacy [11-18], in vitro studies showing the effects of UV on ocular cells are few in number [10,19,20].

The objective of the present study is to develop suitable in vitro methods to detect ocular epithelial cell damage when exposed to UV radiation in an effort to examine UV-absorbing ophthalmic biomaterials. This work involves the exposure of human corneal epithelial cells (HCEC), human lens epithelial cells (HLEC), and retinal pigment epithelial cells (ARPE-19) to UV radiation with and without the protection of UV filters. A UVA and UVB blocking filter (long pass filter [LPF] 400 nm) and a UVB-only blocking filter (LPF 320 nm) were used in this study, to show the protective effects of UV blockers in biomaterials. Cellular viability, mitochondrial dysfunction, DNA damage, and apoptotic activity were analyzed after UV exposure.

## Methods

### Cell culture conditions

Human corneal epithelial cells (HCEC), human lens epithelial cells (HLEC), and retinal pigment epithelial cells (ARPE-19) were prepared, with cultures that were less than 30 passages. Both HLEC and ARPE-19 were obtained from the ATCC, Rockville, MD (American Type Culture Collection; #CRL-11421 and #CRL-2302, respectively), and HCEC were obtained from RIKEN BioResource Center, Tsukuba, Japan (#RCB 2280). The medium used to culture HCEC and ARPE-19 was as follows: 50/50 Ham’s F12/Dulbecco’s modified Eagle’s Medium (Gibco Invitrogen, Grand Island, NY), 10% fetal bovine serum (Gibco Invitrogen), and penicillin/streptomycin (Gibco Invitrogen). The medium used to culture HLEC cells consisted of Minimum Essential Medium, Eagle with Earle’s Balanced Salt Solution (Gibco Invitrogen), 20% fetal bovine serum (Gibco Invitrogen), and penicillin/streptomycin (Gibco, Invitrogen). Cells were incubated in a humidified environment at 37 °C with 5% CO_2_. Cultures were maintained with weekly subculture using the TrypLE Express (stable trypsin replacement; Gibco Invitrogen) and fed every 2 to 3 days.

### Exposure of cells to ultraviolet light

UV filters, both long pass filter (LPF) 400 nm and 320 nm, were obtained from CVI Melles Griot (Albuquerque NM). The cells were transferred into sterile, flat bottom 24-well cell culture plates (BD Falcon, Franklin Lakes, NJ) for the alamarBlue fluorescence measurements, or collagen-coated glass bottom culture Petri dishes (MatTek Corp., Ashland, MA) for confocal scanning laser microscopy. UV exposure was produced by UV fluorescence tubes (Microlites Scientific, Toronto, ON) in a custom designed UV irradiation unit at 37 °C with 5% CO_2_. Before irradiation, the irradiance (W/m^2^) of UV source was calculated with an Instaspec II diode-array spectroradiometer (Oriel Corporation, Stratford, CT), and the calculated irradiance level was 3.9 W/m^2^. In June 1999, the solar ultraviolet irradiance was 2.76 W/m^2^ in Waterloo, ON, Canada [21]. Thus, the levels of artificial UV light used in this study are environmentally relevant. After 24 h of pre-incubation at 37 °C, cells were exposed to UV radiation, with or without UV filter protection, at a distance of 30 cm from the light source for 1, 5, 30, and 60 min (the respective dose was approximately 0.0234, 0.117, 0.702, and 1.404 J/cm^2^). To minimize absorption of the radiation by the medium, a thin layer of medium (about 1.0 mm) was left above the cells during UV exposure.

### AlamarBlue assay

A cell suspension (1 ml) containing 10^5^ cells was seeded in 24-well plates. The plates were then incubated at 37 °C with 5% CO_2_ for 24 h. When the cultures were approximately 75% to 80% confluent, the cells were exposed to UV light. The cultures were then incubated another 24 h at 37 °C with 5% CO_2_. After incubation, the medium was aspirated from each well, and the well was rinsed with 1 ml culture medium without serum. After aspirating the medium, 1 ml of 10% alamarBlue (Invitrogen, Carlsbad, CA) prepared in medium without serum was added to each well. The 24-well culture plate was then incubated at 37 °C for 3 h, and then the fluorescence of each well was determined using a SpectraMax fluorescence multi-well plate reader (Sunnyvale, Ca). Four replicates were used for each treatment. Before the measurements, the excitation/emission wavelengths settings were adjusted to 530/590 nm.

### Hoechst 33342 and rhodamine 123 staining

Confocal scanning laser microscopy (LSM; Carl Zeiss Inc., Toronto, Ontario, Canada) and two fluorescent dyes (Hoechst 33342 and rhodamine 123, Invitrogen) were used to visualize the changes of cell morphologic features (nuclei and mitochondria) after UV radiation. Hoechst 33342 is a popular cell-permeant nuclear stain that emits blue fluorescence when bound to dsDNA [21]. Rhodamine 123 is a cationic dye that stains mitochondria in living cells in a membrane potential-dependent fashion [22]. Before irradiation, 4×10^5^ cells in 1 ml of culture medium were transferred into collagen coated glass bottom culture Petri dishes (MatTek Corporation, Ashland, MA), and grown to confluence at 37 °C with 5% CO_2_ for 24 h. The cultures were then exposed to UV light and incubated for another 24 h. After incubation, the medium was aspirated from each Petri dish, and the dish was rinsed with 1 ml culture medium without serum. After aspirating the medium, the cells were then stained with Rhodamine 123 (20 mM) and Hoechst 33342 (10 mg/ml) for 15 min at 37 °C. After 15 min incubation, the dish was rinsed with 1 ml culture medium without serum once more. A Zeiss confocal laser scanning microscope (CLSM 510; Carl Zeiss Inc.) system attached to an Axiovert 100 microscope with a 40× water-immersion C-Apochromat objective (numeric aperture 1.2) was used to visualize the effects of the two different dyes (n=3 for each treatment). The excitation/emission wavelengths for rhodamine 123 and Hoechst 33342 were 505/534 nm and 355/465 nm, respectively.

### Annexin V staining with LIVE/DEAD Cytotoxicity Assay

Confocal scanning laser microscopy (Carl Zeiss LSM) and three fluorescent dyes (annexin V – Alexa Fluor 647 conjugate, calcein AM, and ethidium homodimer-1; Invitrogen) were used to visualize live, dead, and apoptotic cells after UV exposure. Annexin V stains the cellular membrane of apoptotic cells [23]. Also, calcein AM stains the intracellular cytoplasm of live cells, and ethidium homodimer-1 (EthD-1) stains the nucleic acids of dead cells, respectively [24]. Before irradiation, 4×10^5^ cells in 1 ml of culture medium were transferred into collagen coated glass bottom culture Petri dishes, and grown to confluence at 37 °C with 5% CO_2_ for 24 h. The cultures were then exposed to UV light and incubated for another 24 h. After incubation, the medium was aspirated from each Petri dish, and the dish was rinsed with 1 ml culture medium without serum. After aspirating the rinse medium, the cells were then stained with Annexin V (10 µl in 500 µl buffer), calcein AM (8 µM), and ethD-1 (16 µM) for 15 min at 37 °C. After 15 min incubation, a Zeiss confocal laser scanning microscope (CLSM) 510 system attached to an Axiovert 100 microscope with a water-immersion C-Apochromat objective was used to visualize the fluorescence of three different dyes (n=3 for each treatment). The excitation/emission wavelengths for annexin V, calcein AM, and EthD-1 (in the presence of DNA) were 650/665 nm, 495/515 nm, and 528/617nm, respectively.

### Statistical analysis

For the alamarBlue assay, the statistical significance of differences between treatment groups (four replicates were used for each treatment) was determined using a one-way ANOVA (ANOVA). Pairwise multiple comparison procedures were performed using the Bonferroni posthoc test. Differences were considered significant when the probability was less than 0.05.

## Results

### Calibration of UV light

Before irradiation, the spectral output of the UV source used for the present study was measured with an Instaspec II diode-array spectrometer (Oriel Corporation, CT). The spectral distribution of the UV fluorescent tubes extends from 290 nm to about 370 nm wavelengths, with a peak at around 315 nm. Irradiance measured by the spectrometer was 3.9 W/m^2^, and the radiant exposure (energy level) was determined using the following radiometric equation:

$$\text{H }=\text{ t }\times {\text{ E}}_{ë}$$

where H=radiant exposure (J/cm^2^), t=exposure duration (seconds), and E_ë_=measured irradiance (W/cm^2^). In this study, calculated radiant exposures were 0.0234, 0.117, 0.702, and 1.404 J/cm^2^ for 1 min, 5 min, 30 min, and 1 h exposure duration, respectively.

### Cellular viability

**Figure 1:**
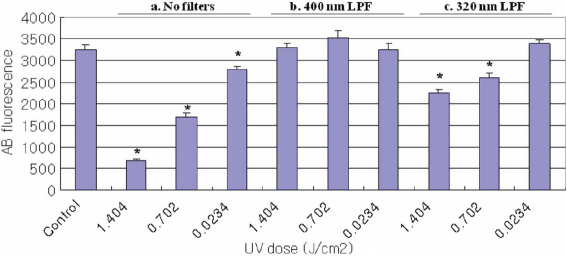
Viability of HCEC Using the AlamarBlue Assay. Cell viability for HCEC irradiated with UV radiation (0.0234, 0.702, and 1.404 J/cm^2^) as revealed by the alamarBlue assay; (a) cell groups without filter protections, (b) cell groups covered with 400 nm LP filters, and (c) cell groups covered with 320 nm LP filters. Significantly lower alamarBlue fluorescence for treated cells compared to control cells (p<0.05) is indicated by an asterisk (*).

**Figure 2:**
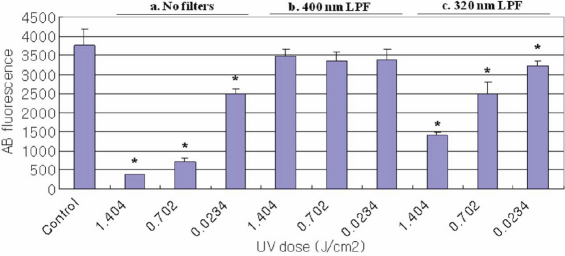
Viability of HLEC Using the AlamarBlue Assay. Cell viability for HLEC irradiated with UV radiation (0.0234, 0.702, and 1.404 J/cm^2^) as revealed by the alamarBlue assay; (a) cell groups without filter protections, (b) cell groups covered with 400 nm LP filters, and (c) cell groups covered with 320 nm LP filters. Significantly lower alamarBlue fluorescence for treated cells compared to control cells (p<0.05) is indicated by an asterisk (*).

**Figure 3:**
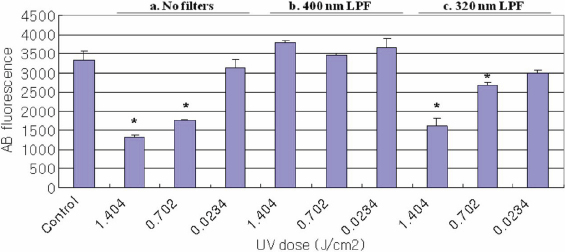
Viability of ARPE-19 Using the AlamarBlue Assay. Cell viability for ARPE-19 cells irradiated with UV radiation (0.0234, 0.702, and 1.404 J/cm^2^) as revealed by the alamarBlue assay; (a) cell groups without filter protections, (b) cell groups covered with 400 nm LP filters, and (c) cell groups covered with 320 nm LP filters. Significantly lower alamarBlue fluorescence for treated cells compared to control cells (p<0.05) is indicated by an asterisk (*).

The effect of UV radiation on change in cell viability as measured using the alamarBlue assay is shown in Figure 1, Figure 2, and Figure 3. The control cultures were not exposed to UV radiation. Cells treated with UV radiation at three different energy levels (0.0234, 0.702, and 1.404 J/cm^2^) without any filter protections, showed dose-dependent decreases in cellular viability. However, 400 nm LPF covered cells treated with UV radiation at three different energy levels did not show decreases in cellular viability. UV-exposed cells with 320 nm LPF protection showed lower cell viability than 400 nm LPF covered cells, yet higher viability than UV-exposed cells without filters, in an energy level-dependent manner. When comparing the three cells exposed with 1.404 J/cm^2^ UV radiation without any filter protection, HLEC showed the lowest cell viability, suggesting that lens epithelial cells may be the most vulnerable to UV radiation.

### Mitochondrial and nucleus morphologies

**Figure 4:**
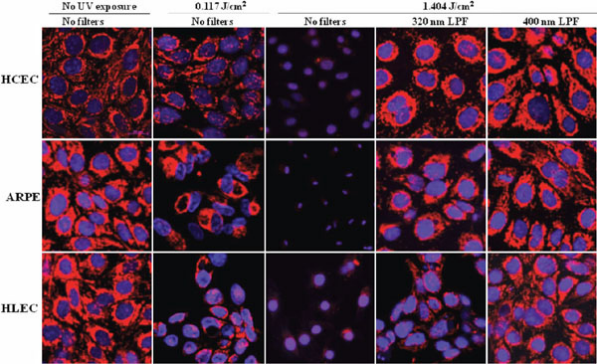
HCEC, HLEC, and ARPE-19 exposed to UV radiation. Representative confocal laser scanning micrographs showing the effect of UV radiation (0.117 and 1.404 J/cm^2^) on distributions of the rhodamine 123 stained mitochondria (red) and Hoechst 33342 stained DNA (blue) in HCEC, HLEC, and ARPE-19 cells.

The effect of UV radiation on change in mitochondrial and nucleus morphologies is shown in Figure 4. The confocal laser scanning micrographs show the distribution of mitochondria (red) and DNA (blue) in the exposed cell lines. The control cells of all three cell lines did not show significant differences in their distribution of mitochondria and DNA. Cells treated with UV radiation at two different energy levels (0.117 and 1.404 J/cm^2^) without any filter protection, showed dose-dependent degradation of mitochondria and DNA. Each cell line treated with 0.117 J/cm^2^ UV without any filter clearly showed reduced mitochondrial and DNA distribution, in comparison to control cells. Furthermore, cells treated with 1.404 J/cm^2^ UV without any filter barely had any mitochondria and exhibited shrunken nuclei. When comparing the three cell lines exposed with 1.404 J/cm^2^ UV radiation without any filter protection, ARPE-19 cells showed the most shrunken nuclei, suggesting that DNA in ARPE-19 cells is possibly the most vulnerable to UV radiation. However, 400 nm LPF covered cells treated with 1.404 J/cm^2^ UV radiation did not show any mitochondrial and DNA damage, showing similar morphology and distribution to control cells. 1.404 J/cm^2^ UV-exposed cells with 320 nm LPF protection showed less mitochondrial distribution than 400 nm LPF covered cells, yet a lot more mitochondrial distribution than UV-exposed cells without filters. The 320 nm LPF covered cells exposed to UV at 1.404 J/cm^2^ did not show substantial nucleic acid damage. This is suggestive that UVB is mostly responsible for DNA damage rather than UVA, as the cells exposed to just UVA (the fourth column in Figure 4) did not show nucleic acid damage, whereas the culture that received UVB and UVA (the third column in Figure 4) showed severe DNA damage.

### Live, dead, and apoptotic cell distribution

**Figure 5:**
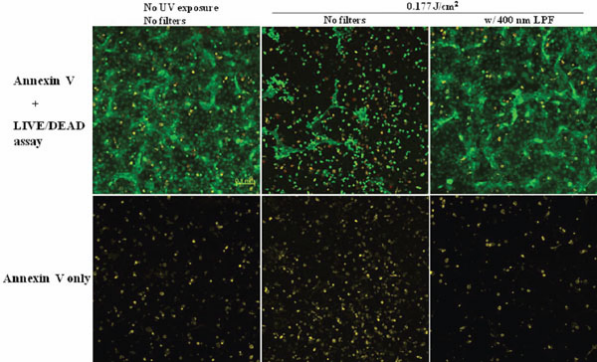
HLEC exposed to UV radiation (@ 0.177 J/cm^2^). Representative confocal laser scanning micrographs showing the effect of UV radiation (0.117 J/cm^2^) on distributions of live (green), dead (red), and apoptotic (yellow) cells in the HLEC cell culture. The first row=annexin V staining with LIVE/DEAD assay, and the second row=annexin V staining only.

**Figure 6:**
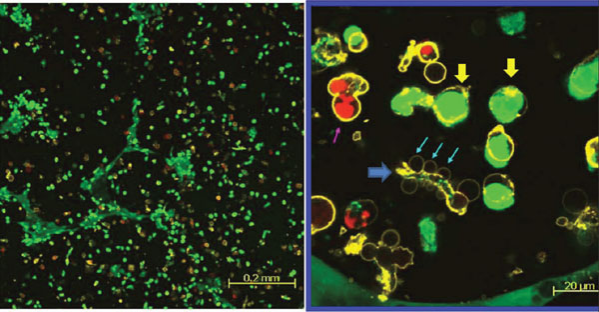
HLEC exposed to UV radiation (@ 0.177 J/cm^2^). Representative confocal laser scanning micrographs showing the effect of UV radiation (0.117 J/cm^2^) on distributions of live (green), dead (red), and apoptotic (yellow) cells in the HLEC cell culture. A closer view of the apoptotic phenomenon; a pink arrow indicates cell blebbing, and blue arrows denote apoptotic bodies. The yellow arrows show cells in early stage apoptosis. The blue arrow shows a cell in a late stage apoptosis.

**Figure 7:**
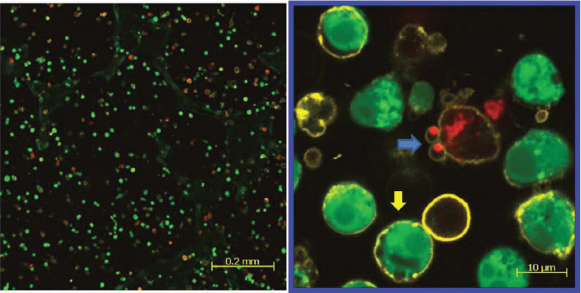
HCEC exposed to UV radiation (@ 0.177 J/cm^2^). Representative confocal laser scanning micrographs showing the effect of UV radiation (0.117 J/cm^2^) on distributions of live (green), dead (red), and apoptotic (yellow) cells in the HCEC cell culture. The yellow arrow shows a cell in early stage apoptosis. The blue arrow shows a cell in late stage apoptosis where the nucleic acids are being localized into apoptotic bodies.

**Figure 8:**
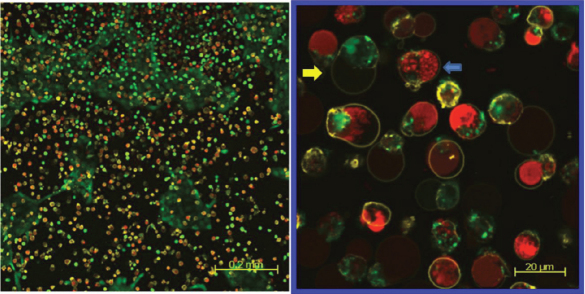
ARPE-19 exposed to UV radiation (@ 0.177 J/cm^2^). Representative confocal laser scanning micrographs showing the effect of UV radiation (0.117 J/cm^2^) on distributions of live (green), dead (red), and apoptotic (yellow) cells in the ARPE-19 cell culture. The yellow arrow shows a cell in early stage apoptosis. The blue arrow shows a cell in late stage apoptosis where the DNA has been fragmented.

The confocal laser scanning micrographs of HLEC stained with calcein AM, ethidium homodimer-1, and annexin V - Alexa Fluor 647 conjugate is shown in Figure 5 and Figure 6. The confocal laser scanning micrographs show the distribution of each live (green), dead (red), and apoptotic (yellow) HLEC. The first row (Figure 5) is the merged images of all three dyes, and the second row only shows the distribution of apoptotic cells. The control showed that most cells were live cells (green) and very few dead cells (red) were present. Some control cells also underwent apoptosis, as a process of natural cell death. However, filter-uncovered cells treated with UV radiation showed an increased number of apoptotic cells as well as dead cells, while filter-protected cells showed no further apoptotic induction. UV-exposed cells also showed a decreased number of live cells in comparison to the untreated control. Figure 6 shows a magnified confocal image of UV-exposed HLEC. Cell shrinkage, cell blebbing (indicated by the pink arrow) and formation of apoptotic bodies (indicated by the blue arrows) were shown. Yellow circled cells with green inside are likely the cells undergoing an early stage of apoptosis, because the intact cell membrane maintains the presence of calcein within the cell. Yellow circled cells with red inside show cells in a later stage of apoptosis, because ethidium homodimer-1 penetrates compromised cell membranes, allowing the binding of Ethidium homodimer-1 to the nucleic acids within the cells. HCEC and ARPE-19 also showed an increased number of apoptotic cells and decreased number of live cells after UV exposure (Figure 7 and Figure 8).

## Discussion

The results of this study demonstrate that UV radiation-induced damage of three different ocular cells in culture (HCEC, HLEC, and ARPE-19) can be evaluated using three assays; the alamarBlue assay, confocal microscopy with rhodamine 123 and Hoechst 33342 staining, and the annexin V staining with LIVE/DEAD Cytotoxicity Assay. Also, the UV blocking efficiency of UV-absorbing interference filters, as alterations of UV-absorbing ophthalmic biomaterials, can be tested using this in vitro assay model. The results clearly revealed that UV radiation can cause decreases in ocular cell viability as well as both DNA and mitochondrial degradations in the three cell lines. In addition, the results showed that UV radiation can also increase the number of apoptotic cells. The 400 nm LP filter was very effective in protecting the cell cultures, as there was no cellular damage at all. However, the 320 nm LP filter-covered cells were damaged to some degree.

There have been many studies that have focused only on showing the spectral transmittance characteristics of various UV- absorbing contact lenses and/or intraocular lenses to verify their anti-UV efficacy [11-18]. However, there are fewer studies showing the cytotoxic effects of UV radiation on ocular cells in terms of cell biology and physiology. It is well known that UV radiation can produce oxidative damage to biomolecules, such as proteins (including enzymes), nucleic acids, and lipids [25,26]. Thus, it can directly impair cellular organelles, including mitochondria, nuclei, and cell membrane in corneal, lens, and retinal cells. Therefore, understanding the cellular and molecular mechanisms of UV-induced ocular cell damage is important to reveal how UV radiation may affect ocular tissue health. In this study, three different ocular cell lines and three different bioassays were used to show UV-induced cellular damage in vitro.

AlamarBlue, also called resazurin, is commonly used as an indicator of chemical cytotoxicity in cultured cells. The assay is based on the ability of viable, metabolically active cells to reduce resazurin to resorufin. This conversion is intracellular, facilitated by mitochondrial, microsomal and cytosolic oxidoreductases [27]. AlamarBlue is non-toxic to cells and stable in culture medium, allowing continuous measurement of cell proliferation in vitro [28] as an endpoint assay. Dose-dependent decreases in the alamarBlue fluorescence readings in this study are due to the loss of appropriate cytoplasmic milieu after UV radiation. For example, free radicals are often generated by UV radiation, and they might have caused the impairment of metabolic enzymes (oxidoreductases) in the cells [29]. In the present study, when comparing the three cell lines exposed to UV radiation without any filter protection, HLEC (Figure 2) showed the lowest and APRE-19 (Figure 3) showed the highest cell viability. This suggests that lens epithelial cells are presumably the most - and retinal pigment epithelial cells the least - vulnerable to UV radiation, among the three cell lines used in this study.

Approximately 90% of the oxygen consumed within a eukaryote is used in mitochondrial respiration, and therefore mitochondria represent the major site for the generation of oxygen-derived free radicals caused by UV radiation [29]. Furthermore, there are obvious relationships between mitochondrial dysfunction and apoptosis [30], so evaluating mitochondrial damage after UV exposure is meaningful. In this study, mitochondrial function was assessed by staining cells with a mitochondrial specific dye, rhodamine 123. Due to the negative potential of mitochondrial inner membrane, cationic rhodamine 123 can only stain mitochondria in living cells in a membrane potential-dependent fashion [22]. Dose-dependent decreases in mitochondrial inner membrane potential after UV exposure are shown in this study (red stain in the first, second, and third column in Figure 4), and these also correspond well with the alamarBlue assay results (Figure 1, Figure 2, and Figure 3). All three cell lines exposed with 1.404 J/cm^2^ UV without filter protection showed only a few mitochondrial residues left (red stain in the third column in Figure 4).

DNA is obviously one of the key targets for UV-induced damage in a variety of organisms, including bacteria [31,32], plants, animals, and humans [33,34]. Therefore, DNA damage after UV exposure was also analyzed in this study using the Hoechst 33342, which is a cell-permeant DNA stain [35]. 0.117 J/cm^2^ UV-exposed cells showed less Hoechst fluorescence, as indicated by dark areas in the nuclei (blue fluorescence of the second column in Figure 4), when compared with the untreated control cells (blue fluorescence of the first column in Figure 4). All three cell lines exposed to UV radiation at 1.404 J/cm^2^ without filter protection exhibited shrunken nuclei (blue fluorescence of the third column in Figure 4). Since it is known that apoptotic cells initially show a reduction in nuclear size and cell volume [35-42], shrunken nuclei found in this study could also be regarded as a sign of early stage apoptosis. On the other hand, the 320 nm LPF-covered cells exposed to UV at 1.404 J/cm^2^ did not show substantial nucleic acid damage (blue fluorescence of the fourth column in Figure 4). These cells were exposed to only UVA radiation. This indicates that UVB is mostly responsible for DNA damage. This finding also corresponds with the fact that UVB directly damages cellular DNA, leading to the formation of pyrimidine dimers [43] and UVA indirectly damages the DNA, via the production of oxygen radical species [43].

Apoptotic activity after UV irradiation was also analyzed in this study using the annexin V staining, along with the LIVE/DEAD Cytotoxicity Assay. These methods were used together to show the distributions of live, dead, and apoptotic cells at once. The LIVE/DEAD Assay kit consists of calcein AM and ethidium homodimer (EthD-1) dyes for detecting live and dead cells, respectively. Non-fluorescent calcein AM is converted into green fluorescent calcein by ubiquitous intracellular esterase activity in live cells [24]. EthD-1 enters cells with damaged membranes and undergoes a 40 fold enhancement of fluorescence upon binding to nucleic acids, thereby producing a bright red fluorescence in dead cells [24]. EthD-1 is excluded by the intact plasma membrane of live cells [24]. Annexin V is a phospholipid-binding protein that has a high affinity for phosphatidylserine (PS), which is located on the cytoplasmic surface of the cell membrane [23]. However, in apoptotic cells, PS is translocated from the inner to the outer surface of the plasma membrane, thus PS is exposed to the external cellular environment [44]. In the present study, cells exposed with UV radiation at 0.177 J/cm^2^ without filter protection clearly showed the induction of apoptosis (yellow stain in the second column in Figure 5), when compared with the 400 nm LP filter protected cells (yellow stain in the third column in Figure 5). Apoptotic changes in the cell include blebbing, loss of cell membrane asymmetry and attachment, cell shrinkage, reduction of nuclear size, nuclear fragmentation, chromatin condensation, and chromosomal DNA fragmentation [36,39,41]. Figure 6 showed some of the apoptotic characteristics, including cell shrinkage, cell blebbing (indicated by a pink arrow) and formation of apoptotic bodies (indicated by the blue arrows). The reduction of nuclear size, another phenomenon of apoptosis, had also been shown earlier using the Hoechst 33342 staining (blue fluorescence of the third column in Figure 4). Incidentally, two different types of apoptotic cells were also shown in Figure 6. The annexin V staining of the PS lipids (yellow ring) is an indication that the PS has translocated from the inner to the outer surface of the plasma membrane. If the cell membrane is still intact, the esterases in the cytoplasm are retained and maintain the green fluorescence of the calcein. In Figure 6, the cells that are green circled by a yellow ring represent early apoptotic cells that have not yet lost plasma membrane integrity. If the cell membrane looses integrity, EthD-1 penetrates the cell and stains the nucleic acid (red). The cells that are red circled by a yellow ring represent late apoptotic cells that have lost their membrane integrity.

In conclusion, the results of this study have shown that cellular viability, mitochondrial function, DNA damage, and apoptotic activity of HCEC, HLEC, and ARPE-19 cells were impaired by environmentally relevant levels of UV radiation in a dose-dependent manner. The three assays (the alamarBlue assay, cell morphology test, and apoptotic activity assay) used to examine ocular cells may offer a sensitive and meaningful biomarker method for predicting the degree of UV-induced ocular cell damage in vitro. Also, this approach and these assays may be of value in future evaluations of UV-absorbing ophthalmic biomaterials.
